# Analyzing the quality differences between healthy and moldy cigar tobacco leaves during the air-curing process through fungal communities and physicochemical components

**DOI:** 10.3389/fmicb.2024.1399777

**Published:** 2024-06-03

**Authors:** Kejian Fu, Xueru Song, Yonghe Cui, Qi Zhou, Yuming Yin, Jilai Zhang, Hongyin Zhou, Youbo Su

**Affiliations:** ^1^College of Resources and Environment, Yunnan Agricultural University, Kunming, China; ^2^Yunnan Tobacco Company Yuxi City Corporation, Yuxi, China; ^3^College of Plant Protection, Yunnan Agricultural University, Kunming, China

**Keywords:** cigar, air-curing, physicochemical properties, mold, microbial communities

## Abstract

**Introduction:**

The air-curing process of cigar tobacco, as a key step in enhancing the quality of cigars, is often susceptible to contamination by mold spores, which severely constrains the quality of cigar tobacco.

**Methods:**

This study employed high-throughput Illumina sequencing technology and a continuous flow analysis system to analyze the differences between the microbial communities and physicochemical components of moldy and healthy cigar tobacco leaves. Furthermore, correlation analysis was performed to reveal the impact of mold on the quality of cigar tobacco.

**Results:**

The differences between the microbial flora and physicochemical compositions of moldy (MC) and healthy (HC) tobacco leaves were analyzed, revealing significant disparities between the two groups. *Aspergillus* spp. represented the dominant mold in MC, with nine out of twelve isolated molds showing higher quantities on MC than on HC. Mold contamination notably decreased the total nitrogen (TN), total phosphorus (TP), total alkaloids (TA), starch, protein, and flavor constituents while increasing the total fatty acid esters (TFAA), which was accompanied by a shift towards weakly acidic pH in the leaves. Fungal community analysis indicated a significant reduction in the fungal operational taxonomic unit (OUT) numbers and diversity indices in MC, contrasting with the bacterial trends. *Aspergillus* exhibited significantly higher relative abundance in MC, with LEfSe analysis pinpointing it as the primary driver of differentiation. Furthermore, significant negative correlations were observed between *Aspergillus* and TP, starch, TA, and protein, while a significant positive association was evident with TFAA. Network analysis underscored the pivotal role of *Aspergillus* as the species influencing disparities between HC and MC, with its abundance serving as a critical determinant during the air-curing process.

**Discussion:**

This study elucidated substantial quality distinctions between MC and HC during air-curing, with *Aspergillus* emerging as the key species contributing to leaf mold.

## 1 Introduction

Tobacco (*Nicotiana tabacum* L.) is a global economic crop extensively cultivated in many countries (Feng et al., 2023). China is the largest tobacco producer, accounting for one-third of the total global consumption. According to the 2021 China Statistical Yearbook, the production of tobacco leaves in China reached 4.59 million tons during the 13th Five-Year Plan period (Dai et al., 2020). As a unique tobacco product, cigars are recognized by their distinct quality characteristics: rich and full-bodied smoke, mellow and abundant aroma, and a flavor that is both bitter and sweet. Additionally, they are perceived as relatively less harmful, which has contributed to the rapid expansion of their consumer market (Yang et al., 2022). Aroma is a critical indicator when evaluating cigar quality. It primarily includes the quality and quantity of the aroma, as well as the fragrance type, which are determined by the variety, content, proportion, and interaction of aromatic components. Characteristics such as a significant amount of aroma, purity, and a distinct fragrance type are general requirements for high-quality cigars (Zheng et al., 2022; Wu et al., 2023). The entire industrial cigar production chain currently presents significant untapped potential, also providing opportunities for creating Chinese-style cigars.

Freshly harvested cigar tobacco leaves are hung in well-ventilated sheds for air-curing, primarily to reduce the excess moisture and bring the leaves to the optimal dryness level for cigar production. During the drying process, various physicochemical changes occur in the leaves, which are crucial for enhancing their final quality (Li N. et al., 2023). First, the color of the leaves gradually changes from fresh green to golden yellow or brown, reflecting the decomposition of chlorophyll and accumulation of carotenoids. Furthermore, natural degradation decreases the nicotine content in the leaves, contributing to a milder cigar taste and reducing their harshness (Jia et al., 2023). Additionally, the conversion of starch and sugar substances during air-curing facilitates a richer aroma and improves the combustion performance of the leaves. The total nitrogen (TN) and total phosphorus (TP) levels are also important indicators for evaluating leaf quality. Nitrogen is a major component of various alkaloids and proteins in tobacco leaves, directly affecting their chemical composition and subsequent sensory quality. The TP content is related to the efficiency of energy conversion and storage in the leaves, influencing their quality after maturation. Rational nitrogen and phosphorus changes during air-curing are essential for developing the desired sensory attributes required for cigars. For instance, the burning rate and aroma release of the leaves can be optimized by adjusting the ratio of these elements, while harmful compounds are gradually reduced during this process (Wang et al., 2009; Sun et al., 2019; Hao et al., 2023). These changes are crucial for enhancing the taste, aroma, and texture of the leaves. Furthermore, after air-curing, the fermented process further enhances the richness and complexity of the taste and aroma of cigar tobacco leaves, which are key factors distinguishing between high-quality and low-quality cigars (Zhao et al., 2022). Therefore, the cigar tobacco air-curing process is critical for improving cigar quality. However, cigar tobacco often suffers from mold contamination during air-curing, severely reducing cigar quality.

During the air-curing process, high temperatures and humidity cause extensive proliferation of mold on the cigar tobacco leaves, which absorbs and decomposes proteins, starches, and other nutrients (Fan et al., 2020). Various microorganisms cause mold in cigar tobacco, including *Aspergillus* spp., *Penicillium* spp., *Rhizopus* spp., *Chaetomium* spp., *Cladosporium* spp., and *Alternaria* spp. Of these, fungi from the *Aspergillus* spp. and *Penicillium* spp. genera, such as *P. citrinum* and *P. chrysogenum* from *Penicillium* spp., and *A. flavus*, *A. niger*, *A. fumigatus*, and *A. polyporicola* from *Aspergillus* spp., represent the primary mold-causing agents in cigar tobacco leaves. *Aspergillus* spp. infestations can cause tobacco leaves to exhibit ecotoxicological characteristics (Fleurat-Lessard, 2017; Ye et al., 2017; Smyth et al., 2019). The green and black pigments released by these molds during infection, along with secreted toxins and the strong moldy odor they produce, affect cigar quality and pose a certain health risk to consumers (Pauly and Paszkiewicz, 2011; Zhou et al., 2024).

Cigar tobacco is susceptible to mold contamination during air-curing, primarily due to the combined effect of various factors, particularly nutrients. The growth of mold pathogens relies on specific nutrients during the cigar tobacco drying process (Zhou et al., 2024). These pathogens mainly include *Aspergillus*, *Penicillium*, and *Fusarium*, which can utilize various organic compounds provided by cigar tobacco as energy and nutritional sources. To obtain energy, mold fungi metabolize carbon sources, such as the sugars (glucose, fructose, and sucrose) in cigar tobacco, and break down other carbon compounds, including cellulose and other polysaccharides. As an essential element for mold growth and enzyme production, nitrogen can be obtained from amino acids, proteins, and their degradation products in cigar tobacco. Studies have shown that mold contamination decreases the nitrogen and phosphorus content in the leaves. These elements are utilized and lost during the metabolic processes of mold microorganisms, especially pathogenic fungal growth (Zhao et al., 2022; Li J. et al., 2023). Plant alkaloids are vital bioactive components of cigar tobacco. Metabolic mold microorganism activities can decompose or transform alkaloids, decreasing the total alkaloid content in tobacco leaves. Furthermore, mold microorganisms can degrade starch and proteins, which reduces their levels in cigar tobacco. Since starch and protein are important precursor materials for cigar tobacco aroma, their reduction or abnormal degradation by molds may significantly impact the product flavor and mouthfeel, reducing its quality and market value (Zhou et al., 2022).

Current research involving the impact of mold on cigar production often focuses on the fermentation stage, with minimal studies available regarding the effect of mold during air-curing. Therefore, this study collected moldy (MC) and healthy (HC) tobacco leaves from cigar tobacco air-curing barns and comparatively analyzed the mold species, microbial communities, and physicochemical components. Redundancy analysis (RDA), correlation heatmaps, and microbial network analysis were used to examine the relationships between these components and the microbial communities. The aim was to identify the main mold species and elucidate the quality differences between MC and HC, providing new insights into the impact of mold during cigar tobacco air-curing.

## 2 Materials and methods

### 2.1 Collection of the cigar tobacco samples

In July 2023, healthy and moldy samples of the Yunxue 39 cigar tobacco leaf variety were collected from the Ede Cigar Tobacco Air-Curing Management Workshop in Mosha Town, Xinping County, Yuxi City, Yunnan Province, China. Two sample groups were established: the healthy cigar tobacco leaf group (HC) and the moldy cigar tobacco leaf group, with a molding rate of ≥80% (MC).

### 2.2 Isolation and identification of pathogenic fungi on the moldy cigar tobacco leaves

The moldy cigar tobacco leaves were crushed using a mortar and pestle, after which the dilution plating method was employed. Here, 1 g of the crushed tobacco sample was added to a test tube, which was filled with sterile water up to the 10 mL mark. The tube was sealed and incubated at 28°C while shaking for 30 min. Then, 1 mL of the supernatant was collected for gradient dilution up to 10^7^. Next, 100 μL samples of the 10^5^, 10^6^, and 10^7^ dilutions were transferred onto 10 different fungal culture media: potato dextrose agar (PDA), Sabouraud dextrose agar (SDA) (Akgül and Çerikçioğlu, 2009), malt extract agar (MEA) (Chapman et al., 2007), modified Leeming and Notman agar (MLNA) (Hsieh et al., 2018), carrot agar medium (CAM), modified Melin-Norkrans medium (MMN) (Vuorinen et al., 2015), fungal isolation medium (FIM) (Arifin et al., 2021), modified Dixon agar (mDixon) (Bond and Lloyd, 1996), panfungal medium (PF), and corn meal agar (CMA) (Walker and Huppert, 1960). Each sample was evenly spread onto these media and incubated at a constant temperature of 30°C for 3 d. The strains of the colonies with different morphologies were collected and purified. The percentage concentrations of the above media are as follows: 3.5% (PDA), 2.4% (SDA), 3.5% (MEA), 2.7% (MLNA), 3.1% (CAM), 2.4% (MMN), 2.7% (FIM), 2.6% (mDixon), 2.5% (PF), 3.5% (CMA).

Using the universal primers ITS1–ITS4, the pathogen’s genomic DNA was amplified through PCR. The forward primer for ITS gene PCR amplification is ITS1 (sequence: CCGTAGGTGAACCTGCGG), and the reverse primer is ITS4 (sequence: TCCTCCGCTTATTGATATGC), with an amplification length of approximately 500 bp. The genomic DNA of the pathogen was extracted using a previously described method (Lõoke et al., 2011). The fungi were obtained via centrifugation at 10,000 × g for 1–3 min. The supernatant was discarded and 0.25 mL LB buffer was added to the sample. After homogenization, the sample was incubated in a 90°C water bath for 15 min, after which 1 μL was transferred to the PCR reaction mixture for amplification. The amplified PCR products were sequenced at Shanghai Rongxu Biotechnology Co., Ltd. and the sequencing results were entered into the National Center for Biotechnology Information (NCBI) database at http://www.ncbi.nlm.nih.gov. The BLASTn tool was used for online comparison with the ITS database of fungal model strains and reference strains. Closely related model fungi were identified based on sequence similarity. A phylogenetic tree was constructed using the Neighbor-Joining method in the MEGA11 software.

### 2.3 Physicochemical composition analysis

The central position of each cigar tobacco leaf sample was selected. For analysis of the total nitrogen (TN), total phosphorus (TP), total alkaloids (TA), amylum, protein, pH, petroleum ether extract (PEE), and total free amino acids (TFAA), the samples were pulverized in liquid nitrogen using a mortar and pestle and stored in an ultra-low-temperature freezer at −80°C. Table 1 lists the methods and equipment used for these determinations (Jia et al., 2023; Ren et al., 2023).

**Table 1:** The methods and equipment used to test the physicochemical composition of the cigar leaves.

Physicochemical composition	Detection methods	Testing instruments and models
Total nitrogen (TN)	Tobacco and tobacco products—determination of the TN. Continuous flow method (YC/T 161-2002)	Continuous flow analyzer AA3
Total phosphorus (TP)	Determination of TP in the plant—Vanadium molybdate blue colorimetric method (NY/T 2421-2013)	UV–Vis Spectrophotometer UV-1200
Total alkaloid (TA)	Tobacco and tobacco products—determination of the TA. Continuous flow (potassium thiocyanate) method (YC/T 468-2013)	Continuous flow analyzer AA3
Amylum	National standard for food safety—determination of amylum in foods (GB 5009.9-2016)	Acid Buret 25 mL
Protein	Tobacco and tobacco products—determination of the protein. Continuous flow method (YC/T 249-2008)	Continuous flow analyzer AA3
pH	Tobacco and tobacco products—determination of the pH (YC/T 222-2007)	pH meter S400-B
Petroleum Ether Extract (PEE)	Tobacco and tobacco products—determination of the PEE (YC/T 176-2003)	Electronic balance (1/10,000) BSA-220.4
Total free amino acid (TFAA)	Tea—determination of the TFAA content (GB/T 8314-2013)	UV–Vis Spectrophotometer UV-1200

The aroma components were analyzed using a gas chromatography-mass spectrometry (GC-MS) system (7890A5975C, Agilent, United States). The cigar tobacco leaves were dried at 40°C for 4 h, ground, and sifted. Next, 20 g of the ground sample was placed in a 1 L round-bottomed flask, after which 350 mL distilled water and 1 mL of a 20 μg/mL internal standard solution (heptadecane) were added. Furthermore, 60 mL of dichloromethane was added to a 100 mL flat-bottomed flask as the extraction solvent, which was installed on a distillation extraction apparatus and heated to boiling point using an electric heating mantle. The extraction flask was heated in a 60°C water bath until the distillation and extraction reached equilibrium, after which extraction was performed for 3 h. The obtained dichloromethane extract was added to 10 g of anhydrous sodium sulfate (Na₂SO₄) and dried overnight. Then, the extract was concentrated and diluted to a final volume of 1 mL, which was transferred to a chromatographic vial for analysis. Based on the precursor substances of the aroma-producing components, the aromatic substances were classified into carotenoid degradation products, Maillard reaction products, chlorophyll degradation products, and cembranoid degradation products (Rodriguez-Bustamante et al., 2005).

### 2.4 Microbial community analysis

The bacterial and fungal diversity in the cigar tobacco leaf samples was analyzed via five biological replicates. For each sample, 100 g of tobacco leaves were ground in liquid nitrogen and divided into three equal parts. Then, the DNA extracted from the three portions of each sample was pooled into one DNA specimen to minimize potential DNA extraction bias. After genomic DNA extraction, its purity was assessed using 1% agarose gel electrophoresis. After PCR amplification and using the preliminary electrophoresis quantification results, the samples were quantified using the QuantiFluor^™^-ST Blue Fluorescence Quantitation System (Promega Corporation). Subsequently, high-throughput sequencing was performed on the Illumina MiSeq platform using a PE (paired-end): 2 × 300 bp sequencing strategy. The V3–V4 hypervariable regions of the bacterial 16S rRNA gene were amplified using the 338F (5′-ACTCCTACGGAGGCAGCAG-3′) and 806R (5′-GGACTACHVGGGTWTCTAAT-3′) primer pair. For the fungal gene, the ITS1F-ITS2R hypervariable regions were amplified using the ITS1F (5′-CTTGGTCATTTAGAGGAAGTAA-3′) and ITS2R (5′-GCTGCGTTCTTCATCGATGC-3′) primer pair. After demultiplexing, the PE reads were subjected to quality control and filtering based on sequencing quality. Subsequently, the reads were merged according to the overlap relationship between the PE reads to obtain optimized data after quality control and merging. Finally, the optimized data were processed using a sequence denoising method (DADA2/Deblur) to acquire the representative amplicon sequence variants (ASVs) and their abundance information. Shanghai Meiji Biomedical Technology Co., Ltd. performed the cigar tobacco leaf DNA extraction, amplification, library construction, sequencing, and data analysis.

### 2.5 Statistical analysis

Single-factor analysis of variance (ANOVA) was used to evaluate the differences between the physicochemical components, microbial diversity, and community compositions of the healthy and moldy cigar tobacco leaves. Pearson correlation analysis was conducted to explore the potential correlations between the fungal communities, fungal diversity, and physicochemical components. The alpha diversity in both groups was examined based on the Sobs, Chao, ACE, Shannon, and Simpson indices to assess the species diversity differences. All calculations and visualizations related to the community analysis were conducted using the R software (version 4.1.1, https://www.r-project.org/) and its “vegan,” “phyloseq,” “DESeq2,” and “picante” packages. Non-metric multidimensional scaling (NMDS) plots based on Bray–Curtis dissimilarity were used to visualize the structural bacterial community changes in the moldy and healthy cigar tobacco leaves. Additionally, the impact of the microbial communities on the physicochemical components of the cigar tobacco leaves was investigated via RDA and correlation heatmaps.

## 3 Results

### 3.1 Isolation and identification of the fungal molds on the moldy cigar tobacco leaves

**Figure 1:**
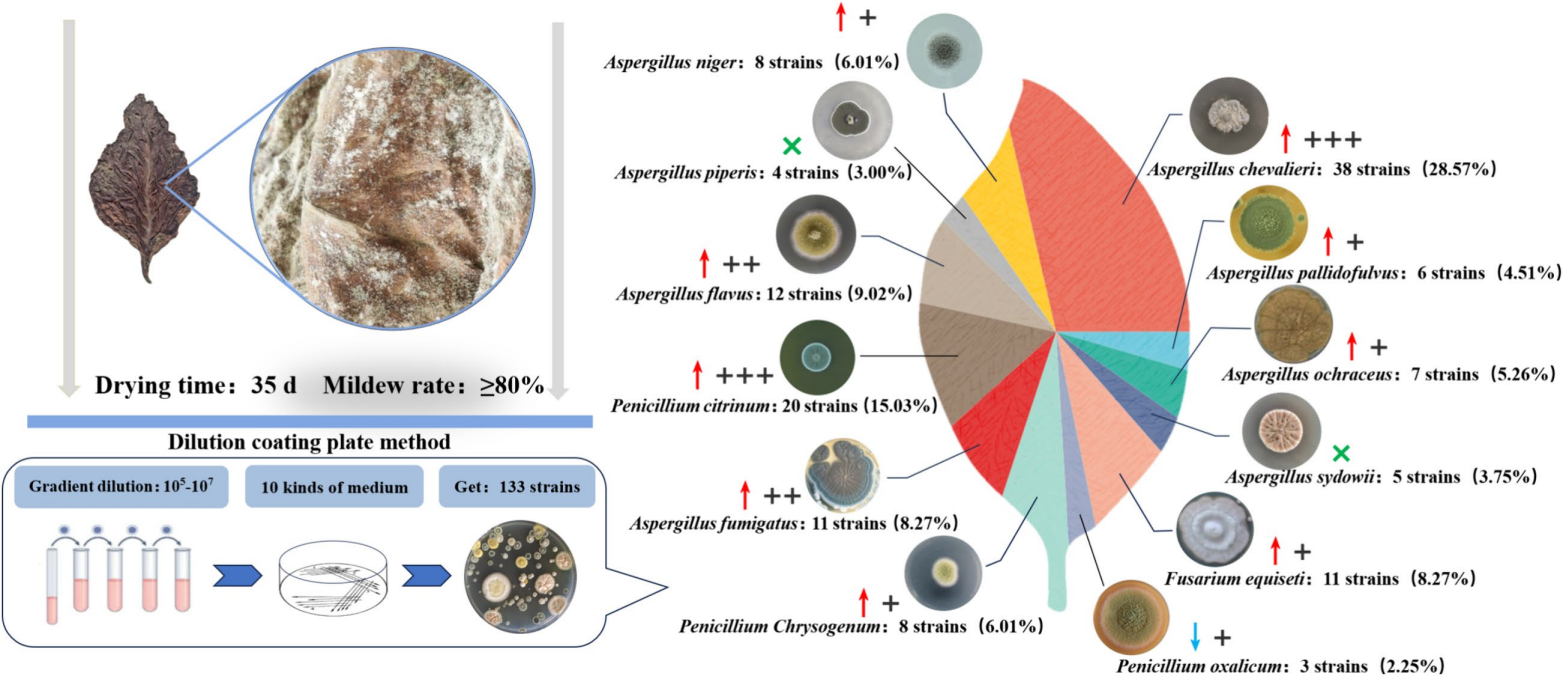
Mold isolation from moldy tobacco leaves. Where “↑” indicates that the number of isolates of this strain is mainly found on the moldy tobacco leaves compared to healthy tobacco leaves, while “↓” indicates that the number of isolates of this strain is lower on the moldy tobacco leaves than the healthy leaves. “×” Indicates that the strains were not isolated on healthy tobacco leaves. + Indicates that the number of excess isolates is between 1–5 strains. ++ Indicates that the number of excess isolates is between 6–10 strains. +++ Indicates that the number of excess isolates exceeds ten strains.

The dilution plating method was used to obtain a total of 133 fungal strains from 10 different fungal culture media based on the colony color and shape differences (Figure 1). Combined with rDNA-ITS sequencing, these were ultimately identified as 12 species belonging to three genera, of which eight species were categorized under *Aspergillus* spp. The 12 fungal species included *A. chevalieri*, *A. pallidofulvus*, *A. ochraceus*, *A. sydowii*, *Fusarium equiseti*, *P. oxalicum*, *P. chrysogenum*, *A. fumigatus*, *P. citrinum*, *A. flavus*, *A. piperis*, and *A. niger*. Nine of these were more frequently isolated from the moldy cigar tobacco leaves than from the healthy leaves, with *A. piperis* and *A. sydowii* absent from the healthy samples. *A. chevalieri* displayed the highest isolation rate of 28.57%, with 21 more strains isolated from moldy cigar tobacco leaves than from the healthy leaves. This was followed by *P. citrinum*, with an isolation rate of 15.03%, and 14 more strains were isolated from the moldy cigar tobacco leaves compared to the healthy ones.

### 3.2 Analysis of the physicochemical component differences between the moldy and healthy cigar tobacco leaves

**Figure 2:**
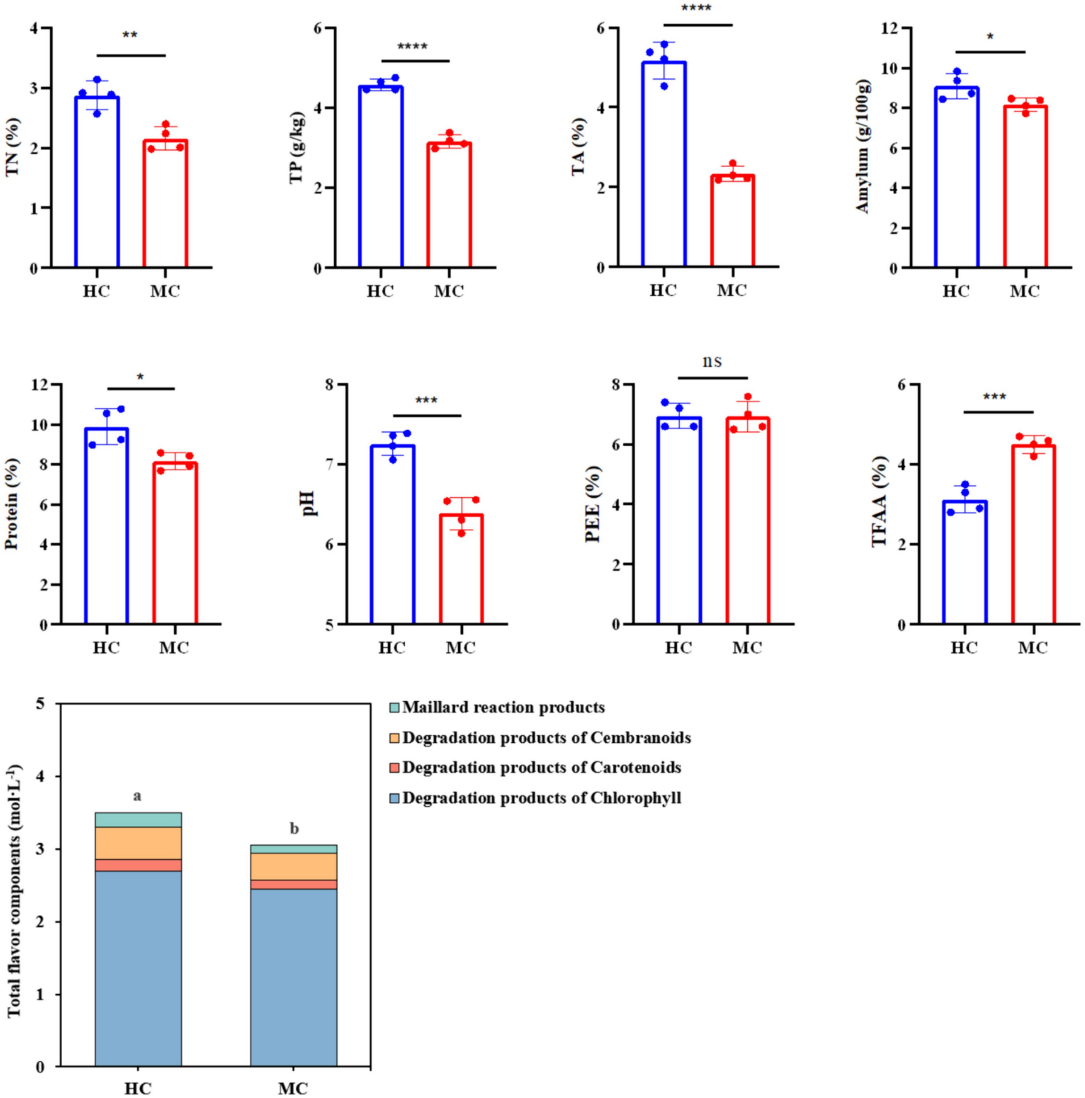
The physicochemical component differences between the healthy and moldy cigar tobacco leaves. According to one-way ANOVA, the number of asterisks indicates the significant differences between treatments: *indicates 0.01 < *p* ≤ 0.05; **indicates 0.001 < *p* ≤ 0.01; ***indicates *p* ≤ 0.001, the same as below.

**Figure 3:**
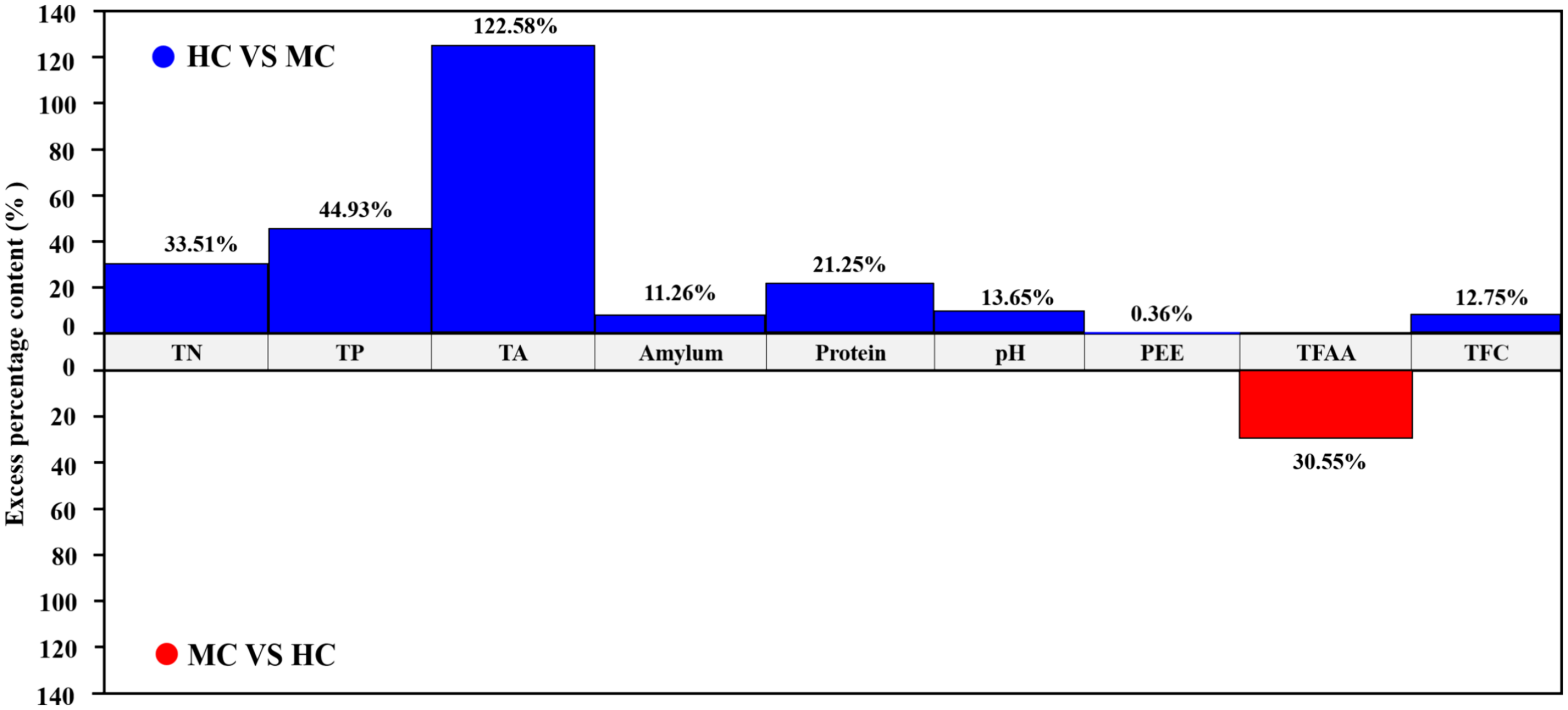
A comparative analysis between the physicochemical components of the healthy and moldy cigar tobacco leaves.

This study compared and analyzed the TN, TP, TA, amylum, protein, pH, PEE, TFAA, and total flavor components (TFC) content in the healthy and moldy cigar tobacco leaves during the air-curing (Figure 2). The moldy cigar tobacco leaf TN, TP, TA, amylum, and protein levels decreased significantly by 33.51, 44.93, 122.58, 11.26, and 21.25%, respectively, compared to the healthy samples (Figure 3). However, the TFAA content in the moldy cigar tobacco leaves was 30.55% higher than in the healthy samples. This may be due to the rapid mold proliferation during spoilage, leading to faster degradation and higher consumption of the TN, TP, TA, amylum, and protein in the moldy cigar tobacco leaves than in the healthy samples. The mold gradually broke down these substances into various amino acids. Additionally, comparing the flavor components indicated that the content of the carotenoid degradation products, Maillard reaction products, chlorophyll degradation products, and cembranoid degradation products was lower in the moldy cigar tobacco leaves than in the healthy leaves, with a total decline of 12.75%. Furthermore, the pH in the moldy leaves was slightly acidic.

### 3.3 Microbial community composition of the moldy and healthy cigar tobacco leaves

**Figure 4:**
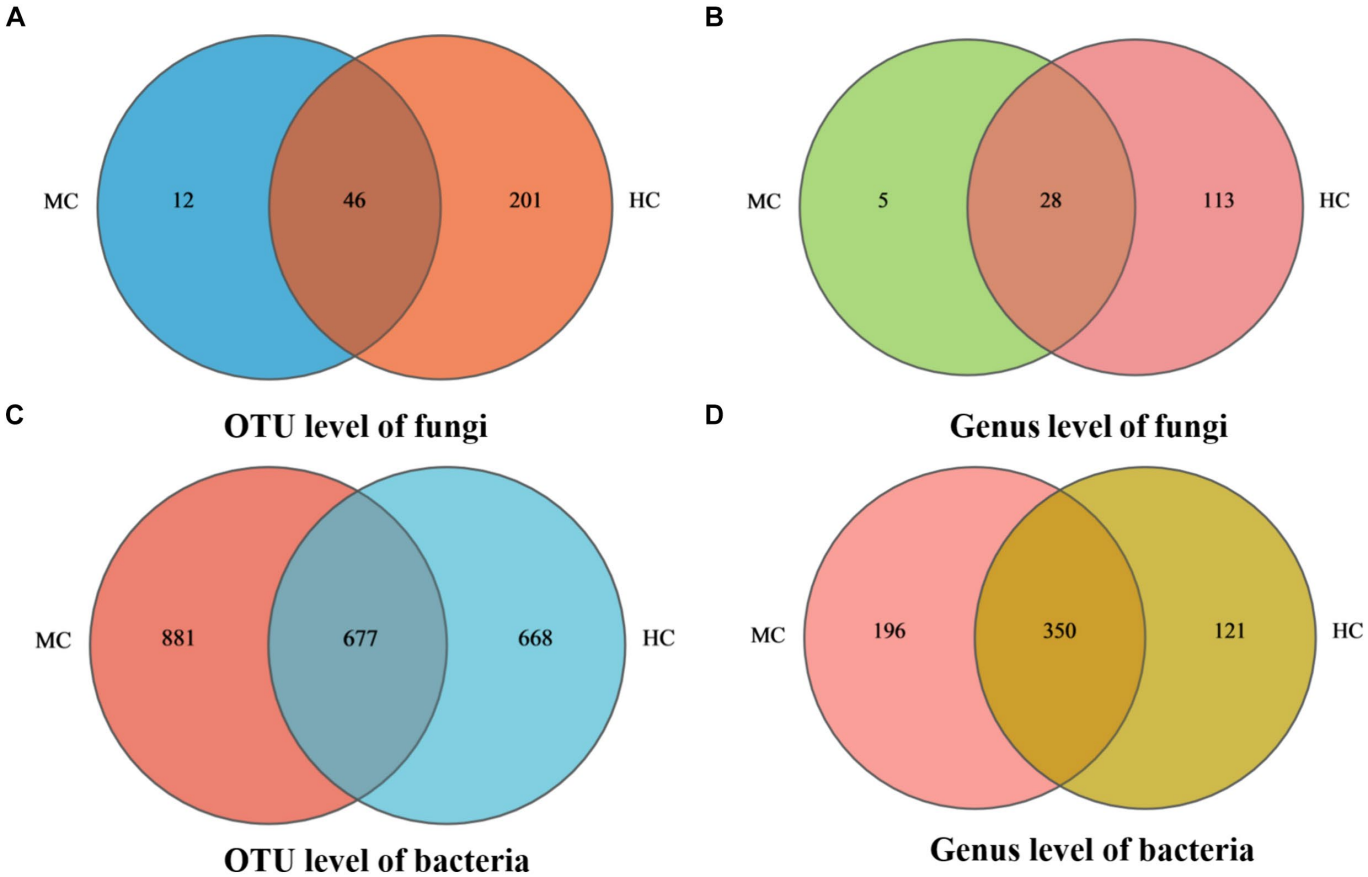
Species Venn diagram. In the figure, **(A)** represents fungal OTU level, **(B)** represents fungal genus level, **(C)** represents bacterial OTU level, and **(D)** represents bacterial genus level.

**Figure 5:**
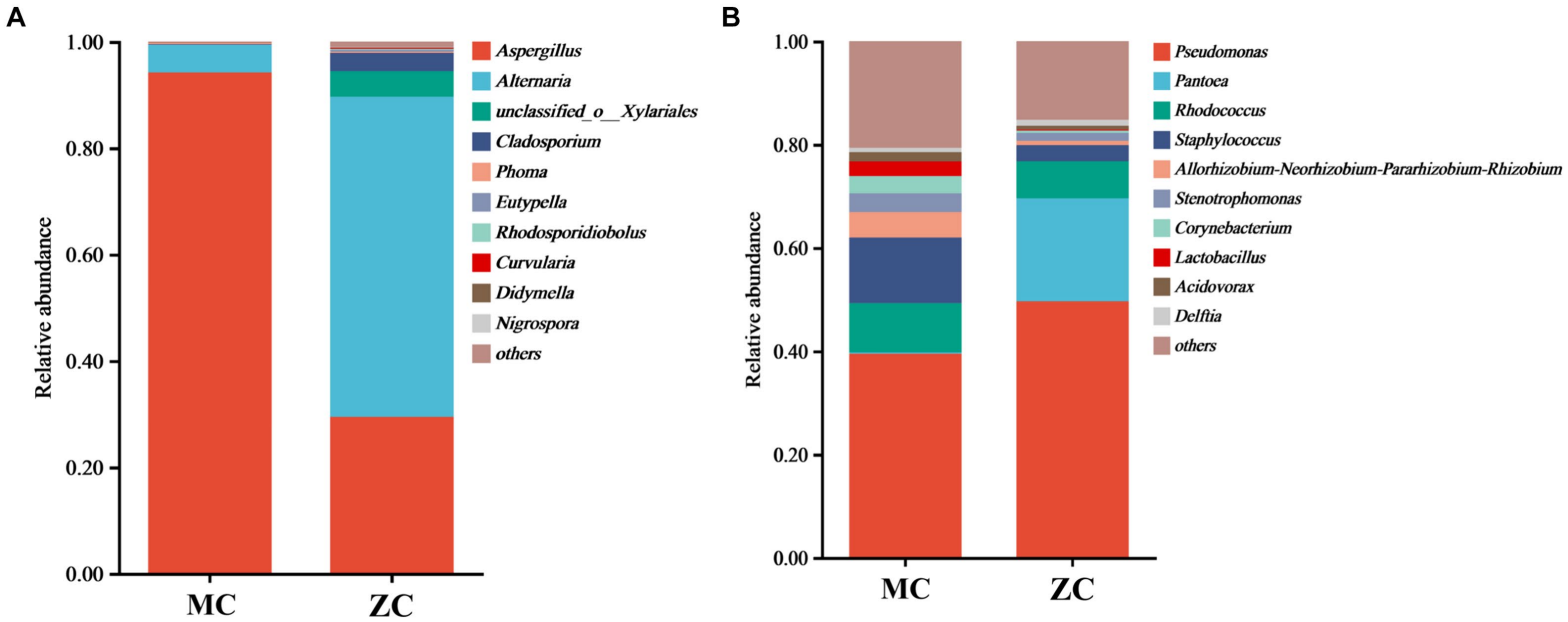
The microbial species abundance of healthy tobacco leaves versus moldy tobacco leaves. In the figure, **(A)** represents fungal genus level; **(B)** represents bacterial genus level.

Venn diagram analysis revealed (see Figure 4) that MC contained a total of 58 fungal operational taxonomic units (OTUs), of which 12 were unique, and 32 fungal genera, of which five were unique. A total of 1,558 bacterial OUTs were identified, of which 881 were unique to MC, while 546 bacterial genera were present, of which 196 were unique. The number of fungal OUTs and genera was significantly lower in MC than in HC, while the bacterial numbers were higher. Furthermore, a comparison between the relative fungal and bacterial abundance at the genus level in MC and HC revealed significant community composition differences. As shown in Figure 5A, *Aspergillus* dominated in MC, with a relative abundance exceeding 60%, which was significantly higher than in HC. Although *Alternaria* and *Cladosporium* were present, their abundance was significantly lower than in HC. Figure 5B shows the relative bacterial genera abundance in HC and MC. *Pseudomonas* dominated in MC with a relative abundance of nearly 40%, while that of *Pantoea* was about 20%. Both belong to the *Proteobacteria* phylum. *Lactobacillus* dominated in HC, with an approximate relative abundance of 30%. It belongs to the *Firmicutes* phylum, a crucial group of lactic acid bacteria commonly used in food fermentation. Acidovorax, also from the *Proteobacteria* phylum, displayed a relative abundance of about 20%. In summary, significant differences were evident between the microbial community compositions MC and HC, with *Aspergillus* and *Alternaria* representing the main differential fungi, which might be key contributors to tobacco deterioration and quality decline. The higher *Cladosporium* and *Lactobacillus* abundance in HC suggests that these microbes play a positive role in maintaining tobacco health. These analyses promote the understanding of the dynamic changes in the microbial ecology of tobacco leaves and can provide a microbiological basis for tobacco quality control.

### 3.4 Microbial diversity index analysis

**Figure 6:**
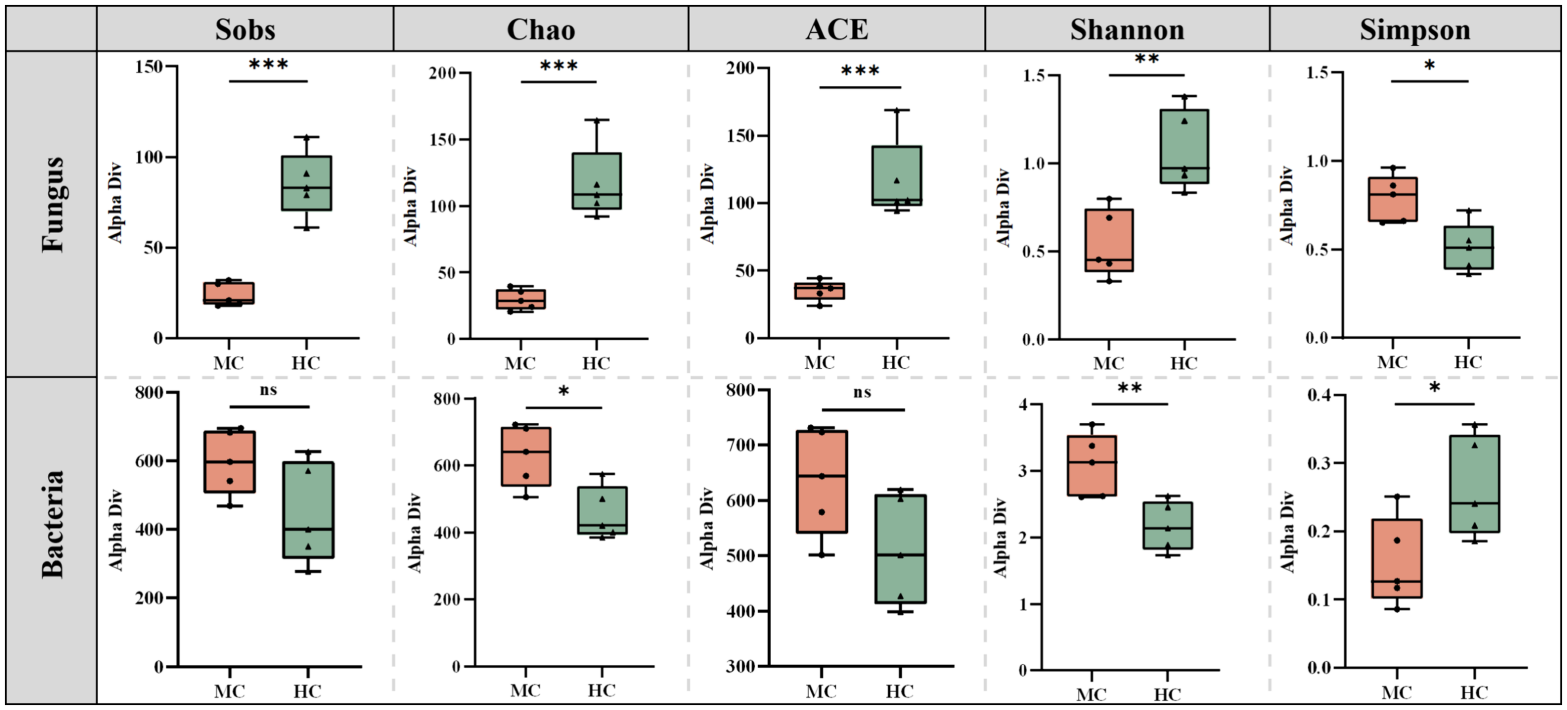
The differences between the microbial diversity changes of the moldy and healthy cigar tobacco leaves.

Mold significantly altered the diversity and richness of the cigar tobacco leaves (Figure 6). The Sobs, Chao, ACE, Shannon, and Simpson indices of the moldy and healthy samples were compared. The results showed that the fungal Sobs, Chao, ACE, and Shannon indices in the moldy leaves were significantly lower than in the healthy leaves, while the Simpson index was considerably higher in the moldy leaves. The bacterial Sobs, Chao, ACE, Shannon, and Simpson indices showed an exact opposite trend to those of the fungi. This suggests that mold can increase the bacterial diversity in cigar tobacco leaves to a certain extent while reducing fungal abundance.

### 3.5 Analysis of the species differences between the moldy and healthy cigar tobacco leaves

**Figure 7:**
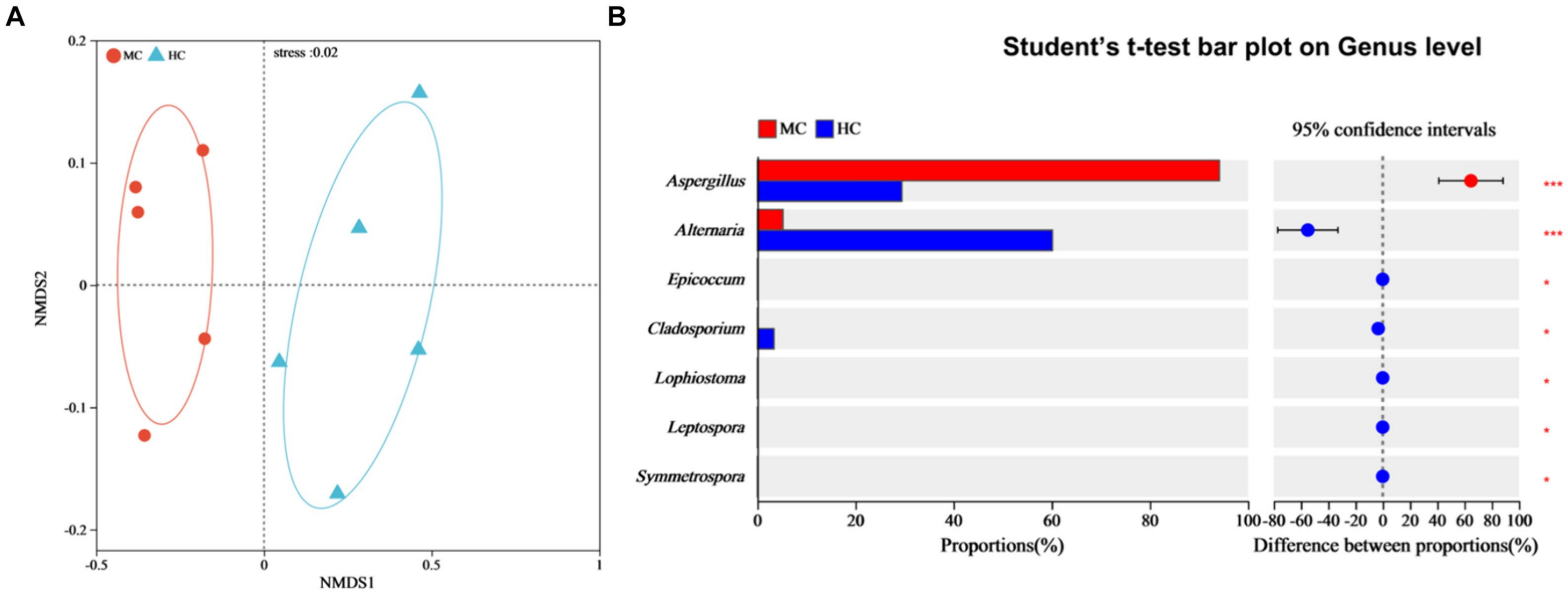
The impact of mold on the fungal community composition of the cigar tobacco leaves. **(A)** The NMDS analysis, where different colors or shapes represent samples from different groups. The closer two sample points are, the more similar their species composition. The horizontal and vertical coordinates represent relative distances and have no actual meaning. Stress: tests the quality of the NMDS analysis results. Generally, if stress <0.2, the two-dimensional plot of NMDS can be used, and its graphic has certain interpretative value. When the stress <0.1, it is considered a good ordination, and when stress <0.05, it is deemed to display very good representativeness. The stress value in the figure is shown with two decimal places. **(B)** Significance testing of the differences between the groups at the fungal genus level.

NMDS analysis was used to compare the dissimilarities between the fungal community compositions of the moldy and healthy cigar tobacco leaves (Figure 7A). Significant differences were evident between the fungal community compositions of the moldy and healthy cigar tobacco leaves (Stress = 0.02), which were tested at the genus level (Figure 7B). The results showed a relative *Alternaria* abundance of 5.22% in the moldy leaves, which was considerably lower than in the healthy leaves, while that of *Aspergillus* was substantially higher at 94.22% (*p* ≤ 0.001). This indicated that *Aspergillus* spp. represented the primary mold-causing fungi in the moldy cigar tobacco leaves, which was consistent with the mold isolation data (Figure 1).

**Figure 8:**
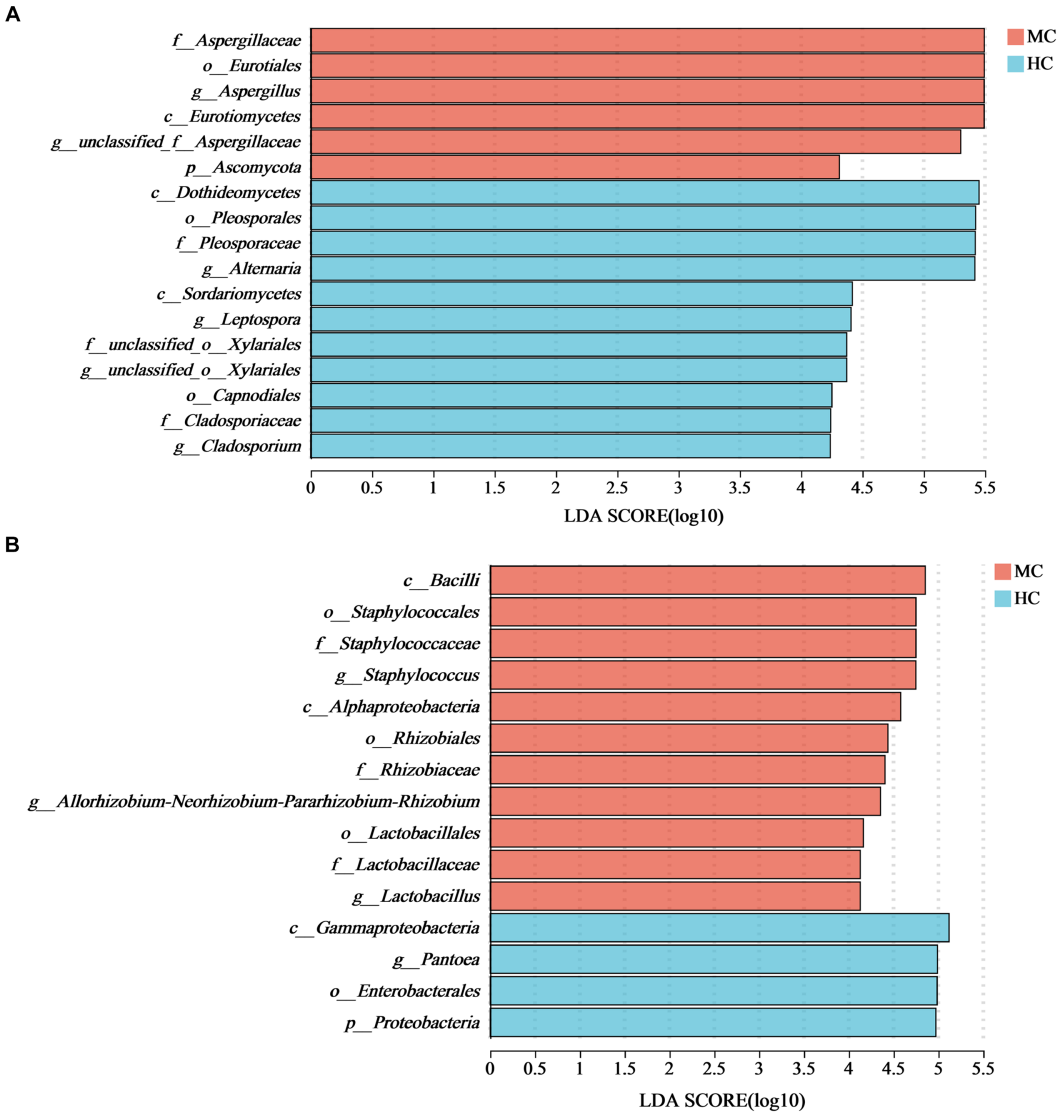
The LEfSe analysis of the microbial communities in the cigar tobacco leaves for **(A)** the fungal communities and **(B)** the bacterial communities. In the vertical axis, the lowercase letters p, c, o, f, and g represent the levels of classification, corresponding to phylum, class, order, family, and genus, respectively.

LEfSe analysis with an LDA threshold of 4 (*p* < 0.05) was used to identify the dominant microorganisms in the moldy and healthy tobacco leaves (Figure 8). The results indicated that the relative abundance of *Ascomycota* and *Proteobacteria* at the phylum level had the most significant impact on the microbial community composition differences between the moldy and healthy cigar tobacco leaves. At the genus level, *Aspergillus* was enriched in the moldy cigar tobacco leaf group, with an LDA score of 5.49, indicating a more substantial impact on the differences. This suggests that *Aspergillus* may play a key role in the molding process of cigar tobacco leaves. Of the bacteria, *Staphylococcus*, *Allorhizobium*-*Neorhizobium*-*Pararhizobium*-*Rhizobium*, and *Lactobacillales* were the dominant microorganisms, which were considered biomarkers to distinguish between moldy and healthy cigar tobacco leaves.

### 3.6 The relationship between the physicochemical properties and biological characteristics of the cigar tobacco leaves

**Figure 9:**
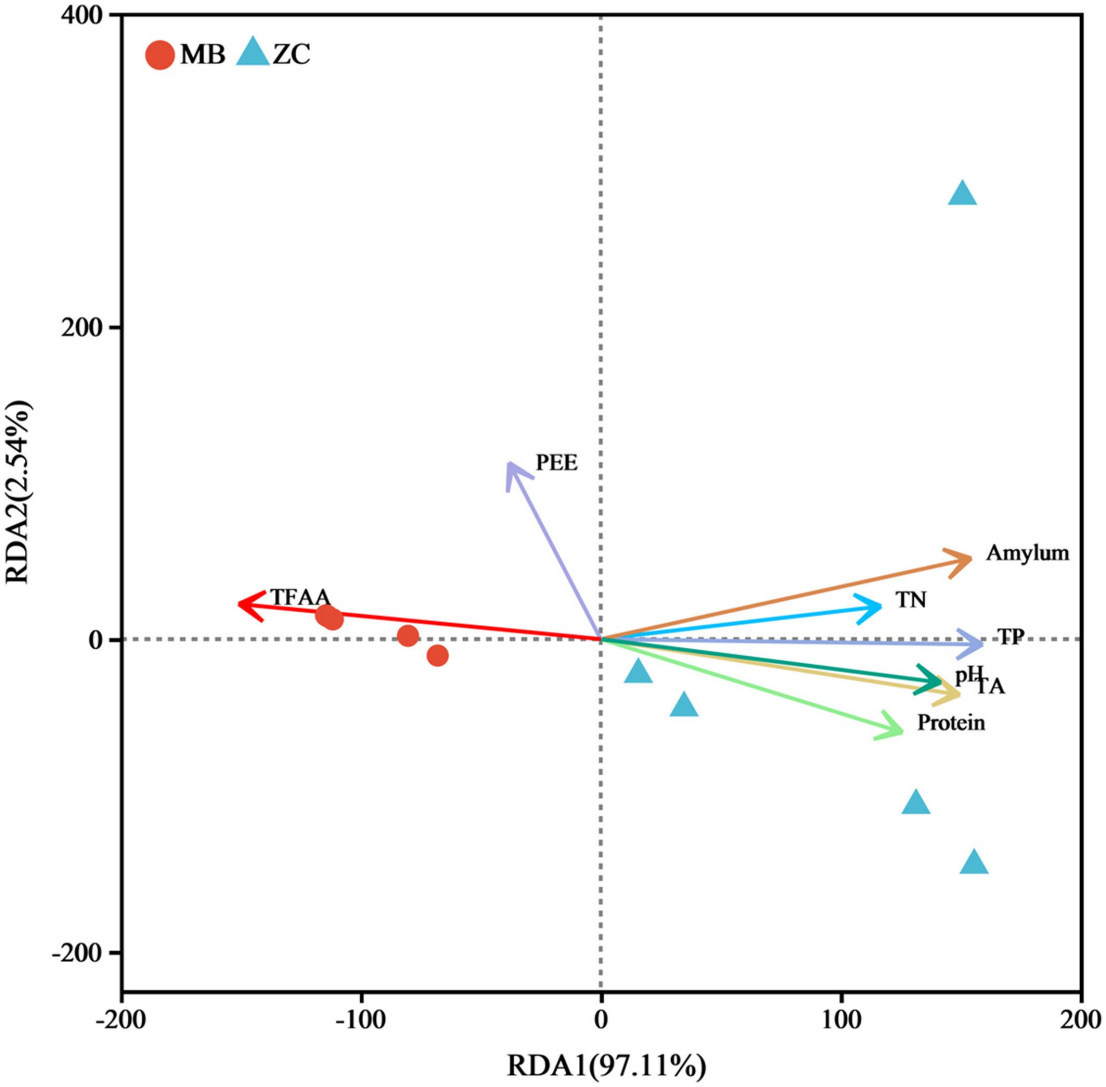
The RDA analysis of the physicochemical properties of the cigar tobacco leaves and fungal communities.

The RDA based on the variance inflation factor (VIF) indicated that TN, TP, TA, amylum, protein, pH, PEE, and TFAA accounted for 99.65% of the total eigenvalues in the fungal community (Figure 9), suggesting that these eight physicochemical properties significantly impacted on the fungal microbiota of the cigar tobacco leaves. The RDA of the physicochemical properties and microbial abundance in the cigar tobacco leaves showed a positive correlation between TFAA and PEE, with TFAA aligning in the same direction as the moldy cigar tobacco leaf samples. This indicated a positive relationship, suggesting that TFAA significantly impacted the fungal microbiome. The amylum, TN, TP, pH, TA, and protein were positively associated with each other and negatively with TFAA and PEE.

**Figure 10:**
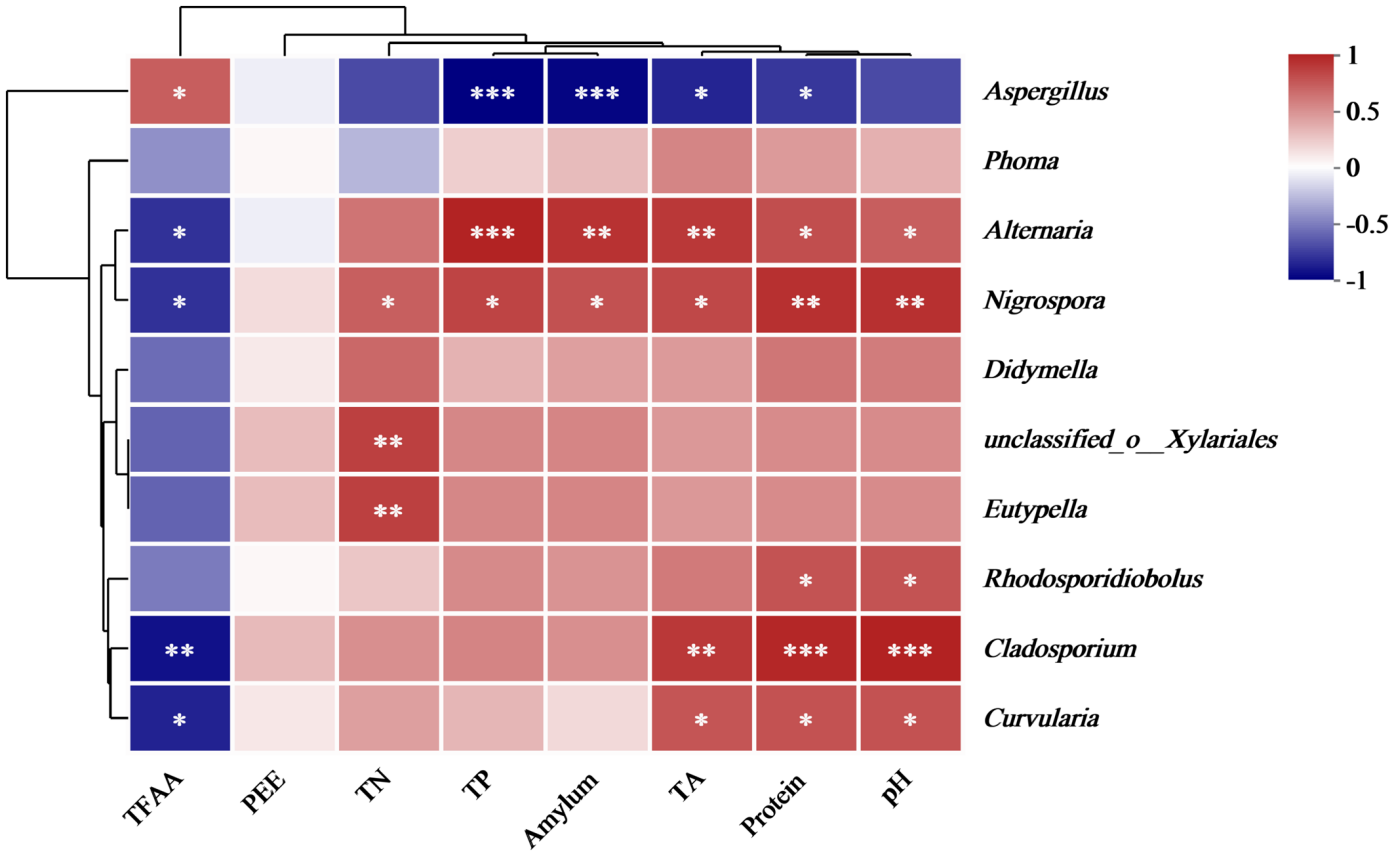
The correlation heatmap of the genus-level fungal communities and fungal diversity with the physicochemical properties of the cigar tobacco leaves. Blue represents a negative correlation, and red denotes a positive association.

Spearman’s correlation analysis was employed to analyze the relationship between the physicochemical properties and fungal microbial abundance in the cigar tobacco leaves. Figure 10 displays the correlation between eight physicochemical properties and 10 key fungal genera at the genus level. *Aspergillus* showed a significant negative correlation with TP, amylum, TA, and protein and a substantially positive correlation with TFAA. Previous results indicated that the main mold pathogen in moldy cigar tobacco leaves is *Aspergillus* spp. represented the main mold pathogen in the MC group, showing a significantly higher relative abundance than in the HC group. This suggests that the mold formation during cigar tobacco air-curing can decrease the TP, amylum, TA, and protein and increase the TFAA level. Furthermore, physicochemical components such as amylum, TA, and protein are crucial precursors of cigar tobacco flavor components. Mold can reduce the content of these three physicochemical components, consequently decreasing the flavor compounds.

### 3.7 Fungal microbial network analysis

**Figure 11:**
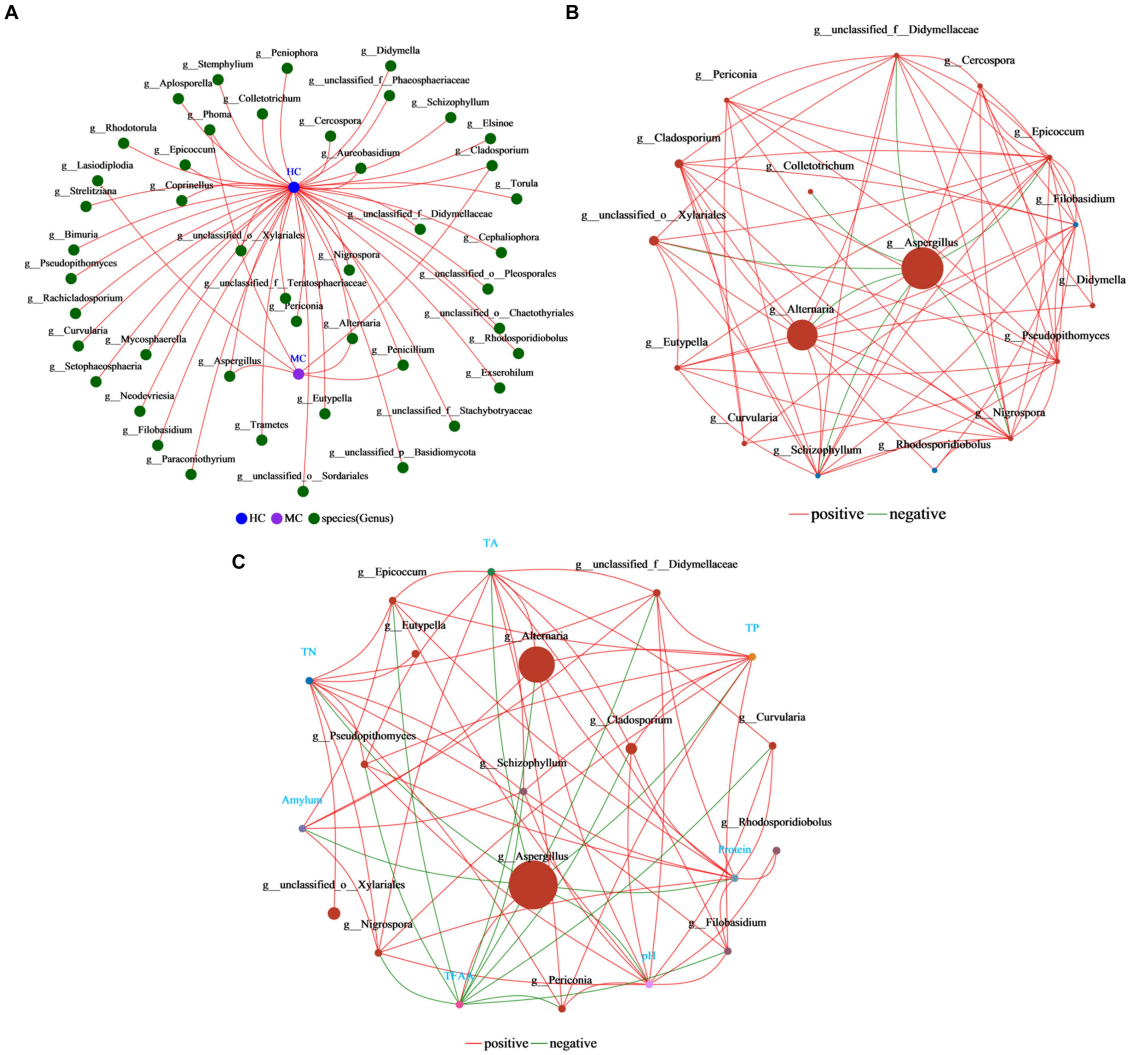
Analysis of the correlation network in the cigar tobacco leaf fungal communities. **(A)** The collinearity network. **(B)** The single-factor correlation network. **(C)** The dual-factor correlation network. The results shown here are all at the fungal genus level. Both the single-factor and dual-factor correlation networks select the top 20 species in terms of overall abundance at the classification level, with correlation coefficients ≥0.5 and *p* < 0.05.

Co-occurrence networks reflect the symbiotic relationships of species in cigar tobacco leaves (Figure 11A). The results showed that the vast majority of the fungi were unique to the healthy cigar tobacco leaves, with only *Lasiodiplodia* being exclusive to moldy leaves. Only *Aspergillus*, *Alternaria*, *Phoma*, *Cladosporium*, and *Penicillium* were common in both the moldy and healthy cigar tobacco leaves, indicating significant differences between the samples. Single-factor correlation networks can be used to analyze the relationships between species (Figure 11B), reflecting the related interactions in cigar tobacco leaves. *Aspergillus* displayed the highest abundance, showing a negative correlation with seven species in the graph and no positive associations. This indicated that the presence of *Aspergillus* affected the survival of other species. Additionally, *Aspergillus* shows correlations with all seven major physicochemical properties, with the only positive association with TFAA (Figure 11C). Overall, these analyses suggest that *Aspergillus* is a key species affecting the differences between healthy and moldy cigar tobacco leaves. Its abundance is critical during cigar tobacco leaf molding.

## 4 Discussion

### 4.1 The main mold-causing fungi during the air-curing process of cigar tobacco

As a unique tobacco product, cigars undergo a manufacturing process different from that of cigarettes. Unlike cigarette tobacco leaves, which are cured by heat, cigar tobacco primarily undergoes three major stages: cultivation and harvesting, air-curing, and fermentation (Shi et al., 2005). Air-curing is the primary process that sets cigars apart from cigarettes. During air-curing, the moisture content of the cigar tobacco leaves is reduced by exposure to air, and variation in this process determines the distinct flavor and aroma characteristics of cigars (Wang et al., 2009; Sun et al., 2019). The temperature and humidity levels required during air-curing are determined based on the moisture levels. Failure to accurately monitor the moisture content during air-curing, along with factors such as high temperatures and humidity, can lead to unfavorable curing conditions and subsequent mold growth (Fan et al., 2020). Fungal organisms, particularly *Aspergillus* species, represent the primary biological factors responsible for mold growth on air-cured tobacco leaves. This study employed ten fungal culture media to isolate mold pathogens, identifying *Aspergillus* spp. as the most abundant. Furthermore, high-throughput sequencing revealed *Aspergillus* as the dominant differential species between moldy and healthy tobacco leaves, with significant enrichment observed in the moldy leaves, suggesting its potential impact on tobacco leaf mold. Studies have shown that *Aspergillus* fungi, known for their wide distribution in various environments, thrive in moldy edible grains, feeds, and stored tobacco leaves. Their significant role in tobacco leaf mold may be attributed to their biological characteristics, such as their environmental adaptability, growth rate, and metabolic product diversity (Park et al., 2005; Hajjaji et al., 2006; Kuiper-Goodman et al., 2010; Senbeta and Gure, 2014; Zhou et al., 2024). Additionally, *A. piperis* and *A. sydowii* were not present on the healthy tobacco leaves, indicating that these fungal species may only grow in specific conditions or may respond to changes in specific environmental factors such as humidity, temperature, and nutrient availability during the molding process (Abarca et al., 2001; Hope et al., 2005). These findings are crucial for understanding the microbial dynamics during the molding process of cigar tobacco leaves. The high isolation rate of specific fungi not only reveals the primary microbial factors during the molding process but also provides clues for further investigating the specific roles of these fungi in tobacco leaf quality deterioration. However, the mold process typically results from the combined action of multiple fungi. While in some cases, a single fungal species (such as *Aspergillus* spp. or *Penicillium* spp.) may play a dominant role in the molding process, in natural environments and storage conditions, mold often involves the interaction of multiple fungi (Magan et al., 2004). These fungi can coexist, compete, or mutually promote each other, collectively influencing the progression and outcome of the molding process. This explains the identification of *Fusarium equiseti* during the isolation process. *Fusarium graminearum Schwabe*, also known as “field fungi,” is a saprophytic and thermotolerant fungus that infects the spikes, grains, and leaves of stressed or injured plants before and after harvest. Cigar tobacco settles on favorable leaf surfaces after harvest, which may be the source of *Fusarium equiseti* in this study (Nelson et al., 1994; Magan and Aldred, 2006; Fleurat-Lessard et al., 2010). Therefore, the complexity of the mold process is not merely attributed to the action of a single type of fungus but rather to the intricate interaction between multiple fungi and environmental factors. However, this study reveals that fungi of the *Aspergillus* genus represent the dominant microbial community responsible for causing mold during the cigar tobacco leaf drying process. Although *Aspergillus* fungi play a pivotal role in cigar tobacco leaf mold, various other fungal species may also be present on the leaf surfaces or in the environment. For instance, this study isolates pathogens such as *Fusarium* spp. and *Penicillium* spp., which may also be involved, even if their effect on tobacco leaves has not been documented by related research. Subsequent in-depth studies may reveal the role of other fungi during the mold process, further supporting the complexity of multiple fungal interactions during mold formation.

### 4.2 The impact of mold on cigar tobacco quality

Mold significantly impacts the quality of cigar tobacco, generally adversely affecting the taste, aroma, safety, and overall enjoyment of cigars (Luo et al., 2015; Yu et al., 2023). During cigar tobacco air-curing in humid environments, the natural fungal spores in the air and curing facilities attach to the leaf surfaces. At optimal temperatures and humidity, the fungi utilize the nutrients in the tobacco leaves to invade the tissue and proliferate extensively (Drachev et al., 2004; Fleurat-Lessard, 2017; Liu et al., 2021; Zhao et al., 2022). The growth of mold pathogens during cigar tobacco air-curing relies on specific nutrients (Schmidt et al., 2019). This study identified the main mold pathogens as species of the *Aspergillus* and *Penicillium* genera. Analysis of the physicochemical composition differences between the moldy and healthy cigar tobacco revealed their ability to utilize various organic compounds as energy and nutritional sources for growth. First, molds primarily metabolize carbon sources for energy. The starch and sugars in cigar tobacco are important carbon sources for molds, which can also degrade other carbon compounds, such as cellulose and other polysaccharides, to obtain energy (Li et al., 2014). Second, nitrogen is essential for mold growth and enzyme production. Amino acids, proteins, and their degradation products can serve as nitrogen sources for molds (Rodgman and Perfetti, 2013). When molds proliferate on cigar tobacco, they consume a large amount of its nutrients, which can directly or indirectly lead to the degradation or transfer of macromolecules, decreasing various physicochemical components in the moldy tobacco leaves. Many of these nutrients are prerequisites for cigar aroma, and their decline affects natural cigar aroma formation and may introduce an unpleasant moldy flavor that masks the original flavor and aroma. Additionally, mold growth can leave visible traces on cigars, such as mold spots or color changes, which can affect the overall appearance and attractiveness of cigars (Welty and Vickroy, 1975; Baraniecki et al., 2002; Maldonado-Robledo et al., 2003). In addition to the overall damage caused by molds, a few species among these “mold pathogens” can produce fungal toxins. For instance, the notorious *A. flavus* produces aflatoxin B1, one of the most potent known natural carcinogens (Klich, 2007; Pauly and Paszkiewicz, 2011). Another mold fungus similar to *A. flavus*, *A. parasiticus*, also primarily produces aflatoxins. In addition to aflatoxin B1, it can also produce other types of aflatoxins such as aflatoxin B2, G1, and G2 (Gourama and Bullerman, 1995). *A. niger* and *A. ochraceus*, known for producing black spores, can produce ochratoxin A in certain conditions, which presents nephrotoxic and carcinogenic properties (Ciegler, 1972; Schuster et al., 2002). Furthermore, some tobacco and respiratory diseases are related to *A. fumigatus*. Although *A. fumigatus* itself is not known as a toxin producer, its presence in improper storage conditions of tobacco or other crops may indicate an environment conducive to the growth of other toxin-producing fungi (Latgé, 2001; Brakhage and Langfelder, 2002; Latgé and Chamilos, 2019). During cigar tobacco air-curing, a wide range of fungal species can colonize the tobacco leaves, with the majority being non-toxin-producing molds. This study isolated several toxin-producing molds, including *A. flavus*, *A. niger*, and *A. ochraceus*, during mold pathogen screening, while others were non-toxin-producing molds. The growth of toxin-producing molds during curing heavily depends on tobacco leaf humidity conditions and the outcome of “ecological niche” competition between a few toxin-producing molds and a large number of non-toxin-producing “storage fungi” species. This competition may be important for effectively allocating resources for promoting accelerated reproduction by toxin-producing molds, making them more likely to occupy more ecological niches (Nelson et al., 1994; Magan et al., 2004; Magan and Aldred, 2008; Fleurat-Lessard et al., 2010). Fungal toxins only form during mold growth. Therefore, when considering preventive controls for safe cigar tobacco air-curing, an overall green mold prevention approach is necessary.

## 5 Conclusion

In summary, this study reveals significant differences between the microbial community structures and physicochemical compositions of healthy and moldy cigar tobacco leaves during air-curing. Compared to healthy cigar tobacco leaves, moldy leaves exhibit a higher number of fungal OTUs but a lower diversity richness. Notably, the bacterial outcomes show an opposite trend. Additionally, the molding process significantly reduces the TN, TP, TA, amylum, proteins, and flavor component content in the cigar tobacco leaves during the TFAA increase. Species difference analysis indicates that the relative abundance of *Aspergillus* reaches 94.22% in the moldy cigar tobacco leaves, which is significantly higher than in healthy leaves. *Aspergillus* shows a notable negative correlation with various chemical components in the cigar tobacco leaves, except for a positive association with the free amino acids content. Moreover, *Aspergillus* spp. is identified as the primary mold-causing fungi in the moldy cigar tobacco leaves. It plays a key role in the quality differences between healthy and moldy cigar tobacco leaves and is crucial for cigar tobacco leaf mold formation. These findings provide a better understanding of how mold affects microbial communities and chemical components during cigar tobacco air-curing. Consequently, this study offers valuable resources for guiding the development of mold prevention strategies during the air-curing process.

## Data availability statement

The datasets presented in this study can be found in online repositories. The names of the repository/repositories and accession number(s) can be found in the article/supplementary material.

## Author contributions

KF: Conceptualization, Data curation, Investigation, Methodology, Validation, Writing – original draft, Writing – review & editing. XS: Project administration, Resources, Writing – review & editing. YC: Project administration, Resources, Writing – review & editing. QZ: Writing – review & editing. YY: Writing – review & editing. JZ: Writing – review & editing. HZ: Writing – review & editing. YS: Formal analysis, Methodology, Supervision, Writing – review & editing.
